# Pre-breeding food restriction promotes the optimization of parental investment in house mice, *Mus musculus*

**DOI:** 10.1371/journal.pone.0173985

**Published:** 2017-03-22

**Authors:** Adam Dušek, Luděk Bartoš, František Sedláček

**Affiliations:** 1 Department of Ethology, Institute of Animal Science, Praha, Czech Republic; 2 Department of Zoology, Faculty of Science, University of South Bohemia, České Budějovice, Czech Republic; Universidade do Porto Instituto de Biologia Molecular e Celular, PORTUGAL

## Abstract

Litter size is one of the most reliable state-dependent life-history traits that indicate parental investment in polytocous (litter-bearing) mammals. The tendency to optimize litter size typically increases with decreasing availability of resources during the period of parental investment. To determine whether this tactic is also influenced by resource limitations prior to reproduction, we examined the effect of experimental, pre-breeding food restriction on the optimization of parental investment in lactating mice. First, we investigated the optimization of litter size in 65 experimental and 72 control families (mothers and their dependent offspring). Further, we evaluated pre-weaning offspring mortality, and the relationships between maternal and offspring condition (body weight), as well as offspring mortality, in 24 experimental and 19 control families with litter reduction (the death of one or more offspring). Assuming that pre-breeding food restriction would signal unpredictable food availability, we hypothesized that the optimization of parental investment would be more effective in the experimental rather than in the control mice. In comparison to the controls, the experimental mice produced larger litters and had a more selective (size-dependent) offspring mortality and thus lower litter reduction (the proportion of offspring deaths). Selective litter reduction helped the experimental mothers to maintain their own optimum condition, thereby improving the condition and, indirectly, the survival of their remaining offspring. Hence, pre-breeding resource limitations may have facilitated the mice to optimize their inclusive fitness. On the other hand, in the control females, the absence of environmental cues indicating a risky environment led to “maternal optimism” (overemphasizing good conditions at the time of breeding), which resulted in the production of litters of super-optimal size and consequently higher reproductive costs during lactation, including higher offspring mortality. Our study therefore provides the first evidence that pre-breeding food restriction promotes the optimization of parental investment, including offspring number and developmental success.

## Introduction

The choice of strategies made by organisms in order to increase their lifetime reproductive fitness is a central issue of life-history evolution [1,2]. Female mammals have evolved a number of state-dependent life-history tactics to increase their fitness prospects [3,4]. An optimal reproductive tactic is defined as a strategy that maximizes an individual’s reproductive value (*i*.*e*. age-specific expectation of all present and future offspring) at every age [5]. A function of an individual’s reproductive value is reproductive effort, reflecting the proportional allocation of available time and energy to reproduction [6]. In female mammals, parental effort (*i*.*e*. total parental investment) typically increases with the resources available [7]. Therefore, in polytocous (litter-bearing) species, litter size is one of the most reliable state-dependent life-history traits that indicate an individual’s parental effort.

David Lack, in his seminal work [8], defined optimal litter size as an average or the most frequent litter size that produces the most offspring able to reproduce. Nevertheless, since the optimal size of the litter can vary through time and space, natural selection may instead operate on parental investment in itself, *i*.*e*. the proportion of parental effort received by each individual offspring [9]. Considering this aspect, Douglas Morris proposed an “optimal investment hypothesis” [10,11] predicting that litter size should increase with an increasing amount of available resources. According to this hypothesis, variation in litter size is a mechanism whereby parents maintain their optimal investment per individual offspring in response to changing environmental or physiological conditions. A competing “individual optimization hypothesis” [12], on the other hand, states that such variation in litter size reflects individual differences in ability to rear offspring.

Parental investment per individual offspring generally increases with decreasing litter size [11,13]. Therefore, natural selection should favour a larger optimal litter size in favourable versus adverse conditions [14,15]. An alternative to this tactic may be to produce larger litters in adverse conditions to compensate for potential offspring loss [16–20]. Because some litters can be smaller (sub-optimal) or larger (super-optimal) than the parental optimum [21,22], parents can also optimize their reproduction either by investing more per individual offspring or by reducing the size of the litter to meet their state-dependent optimum [15]. In addition to this complexity, not only does optimal litter size depend on parental investment in itself, but also on its effectiveness in an individual’s fitness returns. There is a lower threshold below which no offspring can survive and an upper threshold above which an additional investment has no effect on offspring fitness [21]. Therefore, a fundamental question of the optimal investment theory is how much to invest in a particular individual offspring.

In general, there are two basic tactics whereby parents can partition their investment among offspring [11]. If all of their offspring have a similar probability of survival, parents can partition their investment equally. However, if some offspring have a higher probability of survival than others, parents can preferentially favour such competitive offspring or, on the contrary, the marginal offspring with a lower probability of survival. Such preferential investment implies that there might be a parent-offspring conflict [23] and sibling rivalry [24,25]. Therefore, under certain conditions, the optimal investment tactic should be determined by the sum of the fitness of all the family members. For example, if the reduction in the litter size sufficiently enhances the fitness of other family members, the marginal offspring’s inclusive fitness can be best served by dying [26]. An extreme example of this preferential investment can be seen in cases of selective infanticide or selective siblicide of the most vulnerable young (reviewed in [27]).

Selective litter reduction (*i*.*e*. size-dependent natural mortality of the marginal offspring) can operate in all polytocous mammals. Sibling rivalry intensifies as resource availability declines, and thus a reduction of the litter can increase with decreasing availability of resources during lactation [11,15]. Nevertheless, it remains unknown whether and how this tactic is influenced by resource limitations prior to female reproduction. To address this issue, we examined the optimization of parental investment, including litter size, in lactating house mice, *Mus musculus*, exposed to experimental, pre-breeding food restriction (FR). The house mouse is an ideal candidate for investigating this aim for three reasons. First, the reproductive adaptability of the house mouse is probably the most extreme among mammals [28]. Second, female mice regularly face resource limitations in the wild [29]. Third, the litter reduction is a predominant strategy used in this species to stabilize the energy burdens of lactation [30–32].

This study is a follow-up to our previous article [33] in which we investigated the effect of experimental, pre-breeding FR on the sex allocation in female mice. We observed a significantly lower proportion of fertile females in the food-restricted (FR) than in the *ad libitum* (AL)-fed group. However, at the same time, the FR mice tended to produce larger litters (with a higher proportion of daughters) than the AL-fed controls. In addition, we observed that the offspring of the FR mothers suffered substantially less from pre-weaning mortality than those of the AL-fed mothers. Thus, the effect of pre-breeding FR persisted during gestation and even during lactation when we fed both groups *ad libitum*. To understand the mechanisms underlying the optimization of parental investment, involving selective litter reduction, in the present study, we focused on those mouse families (consisting of mothers and their dependent offspring), in which we observed litter reduction (the death of one or more offspring during the lactation period). In the analyses, we used unpublished data from the lactation period together with a subset of our formerly published data [33].

Assuming that pre-breeding food restriction would signal unpredictable food availability, we hypothesized that the optimization of parental investment would be more effective in the FR mice than in the AL-fed controls. First, we checked whether the litter size would vary depending on the pre-breeding feeding treatment and the occurrence of litter reduction. Further, to evaluate fitness optimization, we examined the families with litter reduction for relationships between maternal and offspring condition, and offspring mortality. We predicted that, in comparison to the AL-fed mice, in the FR mice: (1) offspring mortality would be more selective and hence generally lower; and (2) litter reduction would maintain the optimum condition of the mother, and thus would improve the condition and survival of her remaining offspring.

## Materials and methods

### Ethics statement

The animals were cared for in accordance with the principles of the Guide for the Care and Use of Laboratory Animals and the project (Ref. No. 11291/02-30/300) was approved by the Ethical Commission for Animal Experiments of the Ministry of Education, Youth and Sports of the Czech Republic. Animal handling was performed with the utmost care to avoid unnecessary distress. Together with our vet, we regularly checked the health status and mortality of animals throughout the experiment (once a day during the period of intermittent feeding, and at least every fourth day during the lactation period). There were no unexpected deaths of animals. We performed no procedures that would require the use of analgesics and anaesthetics. At weaning (day 21), the mothers and their (21 day old) offspring were euthanized by asphyxiation with carbon dioxide.

### Experimental setup and data collection

We used 152 adult (60–80 day old), sexually mature, virgin, female, outbred CD-1 (ICR) mice (Faculty of Medicine, Masaryk University, Czech Republic). The animals were housed individually in plastic cages (22 × 36 × 14 cm) with wood shavings for bedding, and were supplied with a standard grain-based pellet diet (ST-1, Velas, a. s., Hrabanov, Lysá nad Labem, Czech Republic) and water available *ad libitum*. The breeding females were provided with soft cellulose tissues as nesting material. The colony was maintained in a temperature controlled room (24 ± 1°C) with a 14:10-h light/dark cycle (lights on at 05:00 h).

We collected data in three independent, consecutive, experimental trials. In order to study the effect of pre-breeding resource limitations, 77 randomly selected females were exposed to experimental FR, in the form of the intermittent feeding regime (alternate-day fasting, which consisted in total of four food deprivation and three *ad libitum* 24-h periods), during the week before pairing. For the rest of the experiment, this group of FR females was fed *ad libitum*. In contrast, the AL-fed control females were fed *ad libitum* at all times. At pairing, each female was placed with a randomly selected adult male (60–80 day old; *n* = 152). The males were removed from the cage after 10 days and the females were checked twice daily (at 08:00 and 17:00 h) for delivery. At birth, the pups were counted, individually marked (toe-clipping) and sexed (via a visual inspection of the anogenital region).

**Fig 1:**
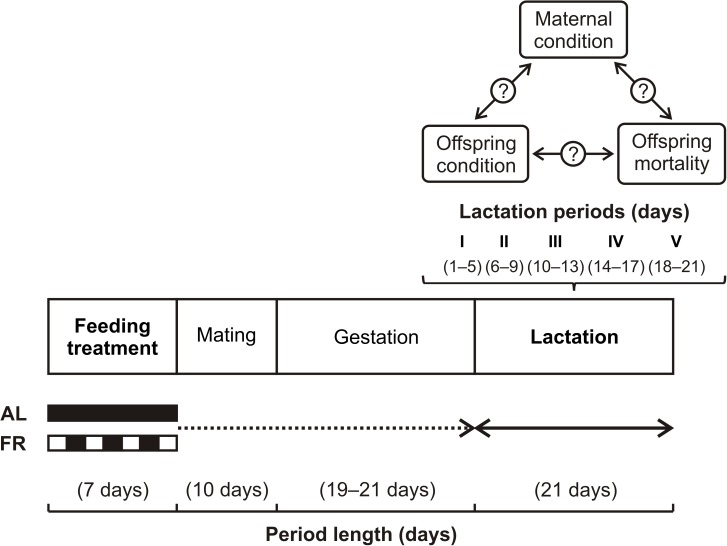
Schematic diagram of the experimental setup. The hypothesized effect of the pre-breeding feeding treatment of the female (*ad libitum*–AL, food restriction–FR) on the optimization of her parental investment during lactation. Considering the changes that may occur with time, we investigated this effect over the course of five equal lactation periods.

During the lactation period, data collection started at birth (day 1) and was performed every fourth day (5, 9, 13, 17, and 21 days of age) until weaning (day 21). On each data collection day, each mother and her remaining offspring were weighed by the same experimenter (AD). Body weight was measured with a digital balance (model EMB 200–2; Kern & Sohn GmbH, Germany; weighing accuracy of 0.01 g). To assess the impact of pre-breeding maternal FR on pre-weaning offspring mortality, we first examined its effect on birth litter size and weaning litter size. In the families with litter reduction (*i*.*e*. the death of one or more offspring from day 1 to 21), we further investigated the effect of pre-breeding maternal FR on total litter reduction (*i*.*e*. the proportion of all offspring deaths from day 1 to 21), and on the relationships between maternal and offspring condition, and offspring mortality (see Fig 1). To account for developmental dynamics (*i*.*e*. actual changes in maternal and offspring weight), we studied fitness optimization, including offspring mortality (*i*.*e*. the proportion of offspring deaths), during five lactation periods (I: day 1–5, II: day 6–9, III: day 10–13, IV: day 14–17, V: day 18–21).

In each period, we assessed (i) maternal condition via the mother’s prior weight and maternal weight change, (ii) offspring condition via the individual offspring prior weight and weight change, and (iii) offspring mortality via the probability of individual offspring death and by litter biomass change. (Both the maternal and offspring prior weights were measured on the last day of a prior lactation period. For lactation period I, we used the data from day 1.) The maternal and individual offspring weight change, and the litter biomass change were computed as:

$$\Delta W=\left(\frac{W_{2}\text{-}W_{1}}{W_{1}}\right)\times 100\;\left(\%\right)$$

where Δ*W* represents the proportional change either of an individual’s body weight (i, ii) or of the litter biomass (iii), and *W*_1_ denotes either an individual’s body weight (i, ii) or the litter biomass (iii) on the first day and *W*_2_ on the last day of a particular lactation period. For all of these variables, the greater the value the better the condition of the individual (i, ii) or the smaller the reduction of the litter (iii).

### Data analysis

All analyses were performed using the SAS System V 9.4 (SAS Institute, Cary, NC; for SAS codes used in the analyses, see S1 Appendix). First of all, we used a *t*-test to evaluate the effect of the feeding treatment on maternal weight at mating (*i*.*e*. on the day after the period of food restriction) and on maternal delivery weight. Further, we aimed to determine the factors that affected birth litter size and weaning litter size. To assess the impact of the feeding treatment on the proportion of all offspring deaths and on the proportions of offspring deaths in particular lactation periods (I–V), we used a *χ*^2^ contingency table analysis. To understand the optimization mechanisms, we examined, from the offspring’s birth to its death or weaning, what factors affected maternal weight change, offspring weight change, and the probability of offspring death.

A General Linear Mixed Model (GLMM) using PROC MIXED was applied in four models to test the fixed effects that affected (a) birth litter size, (b) weaning litter size, (c) maternal weight change, and (d) offspring weight change. We used a Generalized Linear Mixed Model (GzLMM) with PROC GLIMMIX for logistic regression (fitted with a binomial distribution for the error terms and a logit link function) in the fifth model to test the fixed effects that affected (e) the probability of offspring death. The tested fixed effects are listed and defined in Table 1; the models (a–e), in which they were tested, are indicated in the column Analysis. Considering the interplay that exists between maternal and offspring condition, the probability of offspring death was explicitly tested for the interactions between: mother’s prior weight and offspring prior weight; maternal weight change and offspring weight change; mother’s prior weight and offspring weight change; and maternal weight change and offspring prior weight. To assess the impact of pre-breeding maternal FR, we tested all fixed effects, including the interaction terms, nested within the class variable feeding treatment. To account for individual variations among the subjects, we used the random effects experimental trial (a–e) and ID of the mother (d, e). In addition, to account for the repeated measures and genetic relationships, we tested the fixed effects within the class variables lactation period, ID of the mother, and ID of the offspring in the REPEATED statement (c–e). Considering the optimization dynamics (c–e), we evaluated the influence of the fixed effects on the last day of a particular lactation period (I: day 5, II: day 9, III: day 13, IV: day 17, V: day 21). When the offspring died, it was excluded from the analyses (c–e) in subsequent lactation period(s).

**Table 1:** Definition of the Fixed Effects Tested in the Analyses of (a) Birth Litter Size, (b) Weaning Litter Size, (c) Maternal Weight Change, (d) Offspring Weight Change, and (e) Probability of Offspring Death.

Fixed effect	Definition	Analysis
Feeding treatment	Class with two categories (*ad libitum*, food restriction)	a–e
Litter biomass change	Proportional change of litter biomass during a particular lactation period^1^	c, d
Litter reduction	Class with two categories (no, yes)	a, b
Maternal delivery date	Days from pairing to delivery	a, b
Maternal delivery weight	Mother’s weight on day 1	a, b
Maternal weight change	Proportional change of the mother’s weight during a particular lactation period^1^	d, e
Maternal weight change at mating	Proportional change of the mother’s weight during the period of food restriction^2^	a, b
Maternal weight change at weaning	Proportional change of the mother’s weight from day 1 to 21^2^	b
Maternal weight at mating	Mother’s weight on the first day of pairing with a male	a, b
Mother’s prior weight	Mother’s weight on the last day of a prior lactation period^3^	d, e
Offspring sex	Class with two categories (female, male)	c–e
Offspring prior weight	Offspring weight on the last day of a prior lactation period^3^	c, e
Offspring weight change	Proportional change of offspring weight during a particular lactation period^1^	c, e
Prior litter size	Litter size on the last day of a prior lactation period^3^	c–e
Prior litter sex ratio	Proportion of males in the litter on the last day of a prior lactation period^3^	c–e
Secondary sex ratio	Proportion of male births	a, b

^1^Computational method for determining maternal and offspring weight change, and litter biomass change, was described above.

^2^Maternal weight change at mating and at weaning was computed as:
$$\Delta W=\left(\frac{W_{2}\text{-}W_{1}}{W_{1}}\right)\times 100\;\left(\%\right)$$
where Δ*W* represents the proportional change of the mother’s body weight, and *W*_1_ denotes the mother’s body weight on the first day and *W*_2_ on the last day of a particular period studied (the greater the value the better the condition of the mother).

^3^For lactation period I, we used the data from day 1.

To determine the best model, we first included all the terms tested (see Table 1, column Analysis) and then sequentially removed the non-significant ones on the basis of the “Fit Statistics” table, which provides AIC, AICC and BIC information criteria, all in “smaller-is-better” form (as recommended by [34]). We subsequently evaluated the goodness-of-fit of the final models via a visual inspection of the residuals’ normality, the randomness of the error terms, and homoscedasticity. The significance of each fixed effect was assessed by an *F*-test. To evaluate the differences within the class and the significance of each class level, we used a *t*-test. We assessed differences in litter size at birth and weaning among the experimental and control families with and without litter reduction after a Tukey-Kramer adjustment for multiple comparisons. All cited *P*-values were two-tailed, with a significance level (*α*) set at 0.05. To account for an unbalanced design with unequal numbers of AL-fed and FR mothers and their offspring, respectively, the within-class differences were indicated by least-squares (LS)-means (or by logits for GzLMM) with standard errors (± S.E.). We visualized the relationships between the continuous variables by plotting the predicted values of the dependent variable (adjusted for the treatment and covariate effects) against the fixed effect with a predicted regression line.

## Results

In total, 72 of the 75 (96.00%) AL-fed and 65 of the 77 (84.42%) FR female mice littered successfully. Of these, litter reduction occurred in 19 of the 72 (26.39%) AL-fed and in 24 of the 65 (36.92%) FR families. Altogether 56 of the 275 (20.36%) offspring of the AL-fed mothers and 49 of the 351 (13.96%) offspring of the FR mothers died during the lactation period. We observed the loss of the entire litter in 2 of the 19 (10.53%) AL-fed controls.

Pre-breeding FR decreased the weight of the females at mating (means ± S.E.; AL: 27.55 ± 0.33 g; FR: 24.51 ± 0.30 g; *t*_135_ = -6.80, *P* < 0.0001). However, the FR mice compensated for this decrease during gestation; their delivery weights did not differ from those of the AL-fed controls (AL: 38.17 ± 0.37 g; FR: 38.66 ± 0.35 g; *t*_135_ = 0.93, *P* = 0.35). None of the studied variables were affected by maternal weight change at mating, by maternal delivery weight, by maternal delivery date, by offspring sex, by secondary sex ratio, or by prior litter sex ratio (for their use in the models, see Table 1, column Analysis).

### Litter size at birth and at weaning

**Fig 2:**
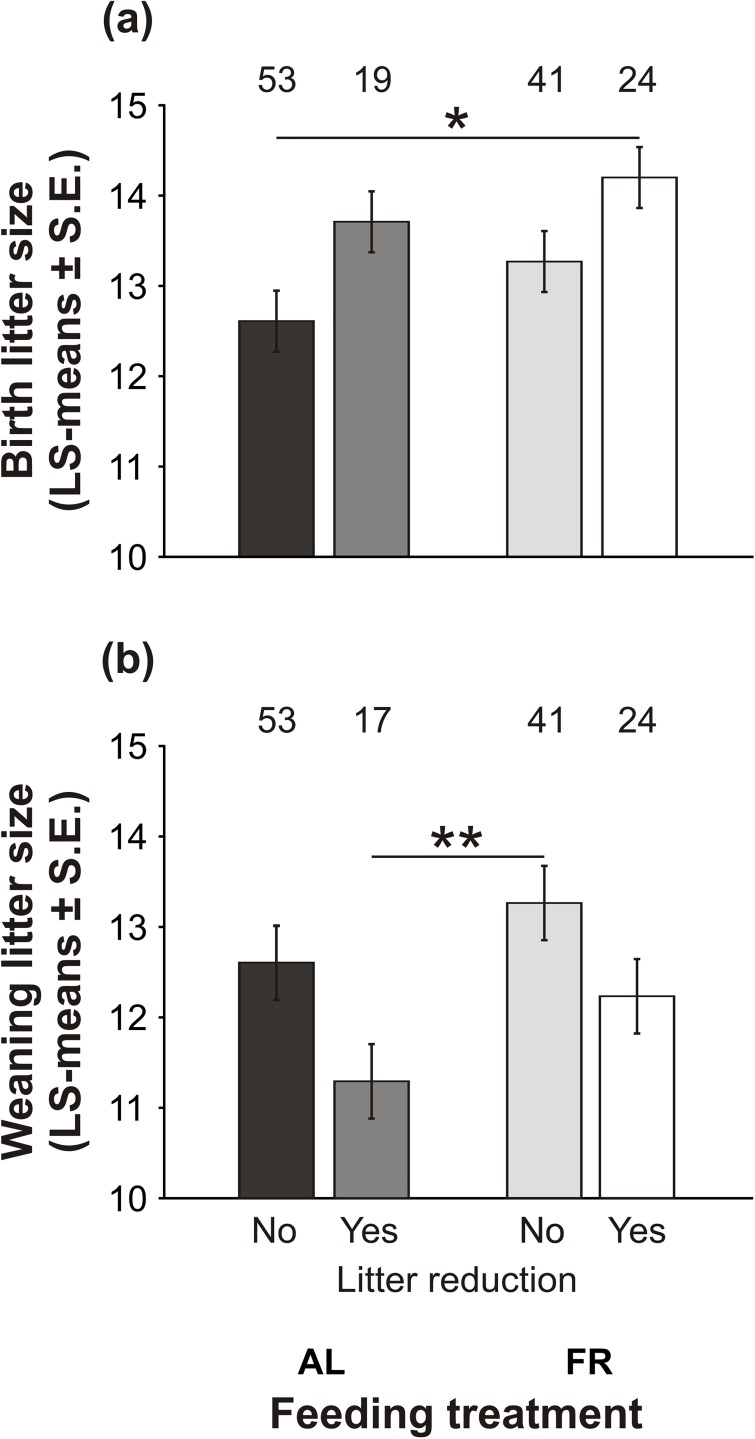
Litter size at birth and at weaning. The effect of pre-breeding maternal feeding treatment (*ad libitum*–AL; food restriction–FR) on litter size (a) at birth and (b) at weaning in the families with and without litter reduction. Bars indicate LS-means ± S.E.; numbers above the bars indicate the sample size (*P < 0.05, **P < 0.01).

The factors that affected birth litter size and weaning litter size are listed in Table 2. On average, birth litter size was smaller in the AL-fed than in the FR mothers (LS-means ± S.E.; AL: 13.16 ± 0.29; FR: 13.74 ± 0.29; Table 2). In both groups, a larger litter size at birth was associated with a later occurrence of litter reduction (Table 2; Fig 2A). We observed the most apparent difference between the FR mice with litter reduction, who produced the largest litters, and the AL-fed controls without litter reduction, who produced the smallest litters (*t*_131_ = 3.15, *P* < 0.02; Fig 2A). Further, the AL-fed and FR mothers differed in birth litter size depending on their weights at mating (Table 2). Whereas birth litter size increased with increasing maternal weight in the AL-fed controls (*t*_131_ = 4.26, *P* < 0.0001), it was independent of maternal weight in the FR mice (*t*_131_ = 0.98, *P* = 0.33).

**Table 2:** Final GLMM Models with Factors that Affected Birth Litter Size and Weaning Litter Size.

Dependent variable	Fixed effect	df (Num, Den)	*F*-value	*P*-value
Birth litter size	Feeding treatment	1, 131	4.54	< 0.05
	Litter reduction (Feeding treatment)	2, 131	4.09	< 0.02
	Maternal weight at mating (Feeding treatment)	2, 131	9.56	< 0.0001
Weaning litter size^1^	Feeding treatment	1, 127	4.39	< 0.05
	Litter reduction (Feeding treatment)	2, 127	4.95	< 0.01
	Maternal weight at mating (Feeding treatment)	2, 127	9.90	< 0.0001
	Maternal weight change at weaning (Feeding treatment)	2, 127	9.47	< 0.0001

^1^Two AL-fed controls were excluded from this analysis due to the loss of their entire litters.

The effect of pre-breeding FR on litter size persisted until weaning, despite the reduction of some litters during lactation. On average, weaning litter size was smaller in the AL-fed than in the FR mothers (AL: 11.94 ± 0.31; FR: 12.74 ± 0.30; Table 2). In both groups, litter reduction contributed to the variation in litter size at weaning (Table 2; Fig 2B). The FR mice without litter reduction weaned the largest litters, which substantially varied from the AL-fed controls with litter reduction, who weaned the smallest litters (*t*_127_ = 3.22, *P* < 0.01; Fig 2B). Similarly as at birth, pre-breeding FR also influenced the relationship between maternal weight at mating and litter size at weaning (Table 2). Weaning litter size increased with increasing maternal weight in the AL-fed controls (*t*_127_ = 4.31, *P* < 0.0001), whereas it was independent of maternal weight in the FR mice (*t*_127_ = 1.12, *P* = 0.26). On the other hand, in both groups, we found a positive association between maternal weight change and litter size at weaning (Table 2). We observed larger litters in the mice that had gained weight during lactation than in those that had lost weight.

### Litter reduction over lactation

**Fig 3:**
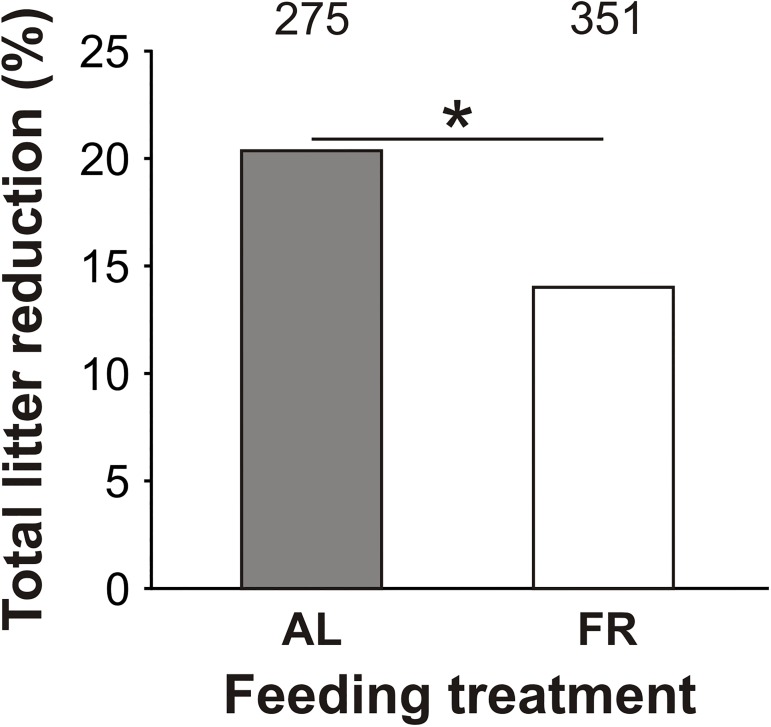
Total litter reduction. The effect of pre-breeding maternal feeding treatment (*ad libitum*–AL; food restriction–FR) on total litter reduction (proportion of all offspring deaths from day 1 to 21); numbers above the bars indicate the sample size (*P < 0.05).

In the families with litter reduction, we observed that total litter reduction (proportion of all offspring deaths from day 1 to 21) was higher in the AL-fed than in the FR mice (*χ*^2^_1_ = 4.53, *P* < 0.05; Fig 3).

**Fig 4:**
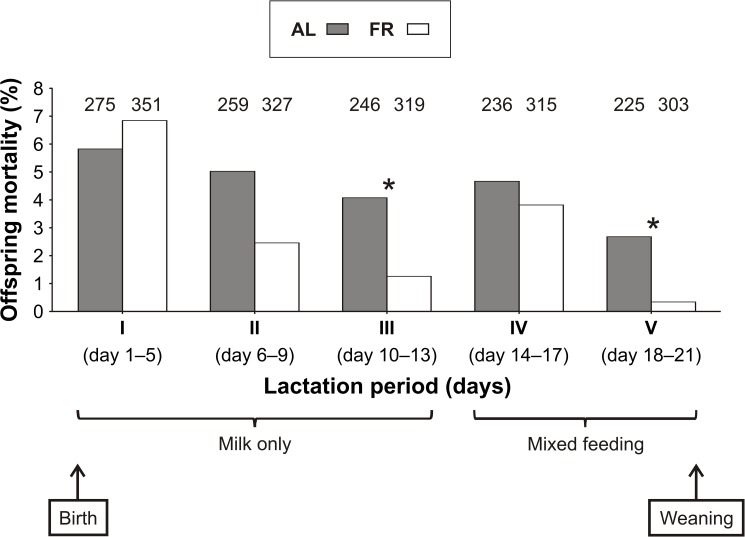
Dynamics of offspring mortality. The effect of pre-breeding maternal feeding treatment (*ad libitum*–AL; food restriction–FR) on the proportions of offspring deaths in particular lactation periods; numbers above the bars indicate the sample size (*P < 0.05).

In both groups, the mortality of the young declined with age (Fig 4). With exception to lactation period I, when offspring mortality (proportion of offspring deaths) was slightly lower in the AL-fed than in the FR group, there was a consistent trend for higher offspring mortality in the AL-fed than in the FR group in subsequent (II–V) lactation periods (Fig 4). However, this difference was only significant for lactation periods III (*χ*^2^_1_ = 4.54, *P* = 0.03; Fig 4) and V (*χ*^2^_1_ = 5.39, *P* = 0.02; Fig 4).

### Fitness optimization in families with litter reduction

#### Maternal weight change

**Fig 5:**
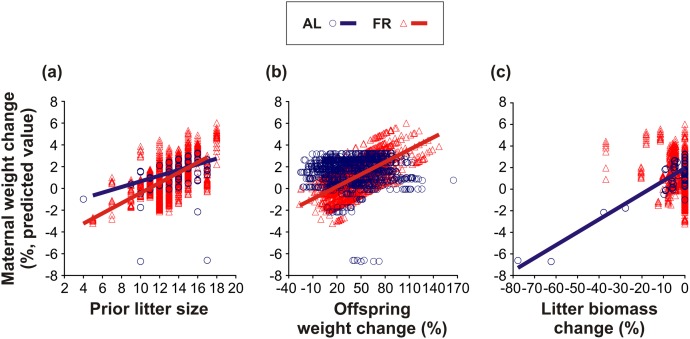
Maternal weight change. The effect of pre-breeding maternal feeding treatment (*ad libitum*–AL; food restriction–FR) on the relationships between maternal weight change and (a) prior litter size, (b) offspring weight change, and (c) litter biomass change. Only the regression lines of the significant relationships are shown.

The factors that affected maternal weight change are listed in Table 3. When comparing the groups, the AL-fed mothers, on average, gained more weight than the FR mothers (AL: 1.40 ± 0.54%; FR: 0.84 ± 0.54%; Table 3). In both groups, maternal weight change was positively associated with prior litter size (Table 3; Fig 5A). However, this relationship was less pronounced in the AL-fed than in the FR mothers (AL: *t*_420_ = 6.99, *P* < 0.0001; FR: *t*_536_ = 11.44, *P* < 0.0001; Fig 5A). Furthermore, the weight change of the AL-fed and the FR mothers differed according to the weight change of their offspring (Table 3; Fig 5B). Whereas the weight change of the AL-fed mothers was independent of their offspring’s weight change (*t*_1231_ = 0.10, *P* = 0.92; Fig 5B), the weight change of the FR mothers was positively associated with the weight change of their offspring (*t*_1608_ = 4.82, *P* < 0.0001; Fig 5B). In addition, the weight change of the AL-fed and the FR mothers differed depending on litter biomass change (Table 3; Fig 5C). Whereas the AL-fed mothers lost weight along with the reduction of the litter (*t*_790_ = 4.89, *P* < 0.0001; Fig 5C), the FR mothers did not show such a tendency (*t*_768_ = -0.12, *P* = 0.90; Fig 5C).

**Table 3:** Final GLMM Model with Factors that Affected Maternal Weight Change.

Fixed effect	df (Num, Den)	*F*-value	*P*-value
Feeding treatment	1, 776	21.54	< 0.0001
Prior litter size (Feeding treatment)	2, 472	90.56	< 0.0001
Offspring weight change (Feeding treatment)	2, 1401	11.63	< 0.0001
Litter biomass change (Feeding treatment)	2, 779	11.98	< 0.0001

#### Offspring weight change

**Fig 6:**
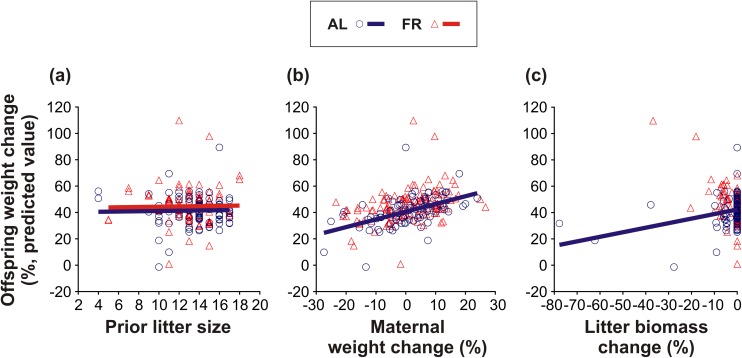
Offspring weight change. The effect of pre-breeding maternal feeding treatment (*ad libitum*–AL; food restriction–FR) on the relationships between offspring weight change and (a) prior litter size, (b) maternal weight change, and (c) litter biomass change. Only the regression lines of the significant relationships are shown.

The factors that affected offspring weight change are listed in Table 4. Unlike their mothers, the offspring of the AL-fed mothers, on average, gained less weight than those of the FR mothers (AL: 45.24 ± 6.07%; FR: 53.23 ± 5.29%; Table 4). In both groups, offspring weight change was positively associated with the prior litter size of the mother (Table 4; Fig 6A). This relationship was, however, slightly less pronounced in the offspring of the AL-fed mothers in comparison to the offspring of the FR mothers (AL: *t*_2254_ = 4.24, *P* < 0.0001; FR: *t*_592_ = 10.72, *P* < 0.0001; Fig 6A). Moreover, in both groups, offspring weight change also tended to be positively associated with maternal weight change (Table 4; Fig 6B). Nevertheless, this association was only significant in the AL-fed group (AL: *t*_2227_ = 6.42, *P* < 0.0001; FR: *t*_2198_ = 0.56, *P* = 0.57; Fig 6B). In addition, the groups radically differed in offspring weight change depending on litter biomass change (Table 4; Fig 6C). Along with the reduction of the litter, the remaining offspring of the AL-fed mothers lost weight (*t*_1206_ = 6.10, *P* < 0.0001; Fig 6C), whilst the remaining offspring of the FR mothers did not show such a trend (*t*_1571_ = 0.94, *P* = 0.35; Fig 6C).

**Table 4:** Final GLMM Model with Factors that Affected Offspring Weight Change.

Fixed effect	df (Num, Den)	*F*-value	*P*-value
Feeding treatment	1, 264	37.52	< 0.0001
Prior litter size (Feeding treatment)	2, 1034	66.46	< 0.0001
Maternal weight change (Feeding treatment)	2, 2232	20.75	< 0.0001
Litter biomass change (Feeding treatment)	2, 1373	19.02	< 0.0001

#### Probability of offspring death

**Fig 7:**
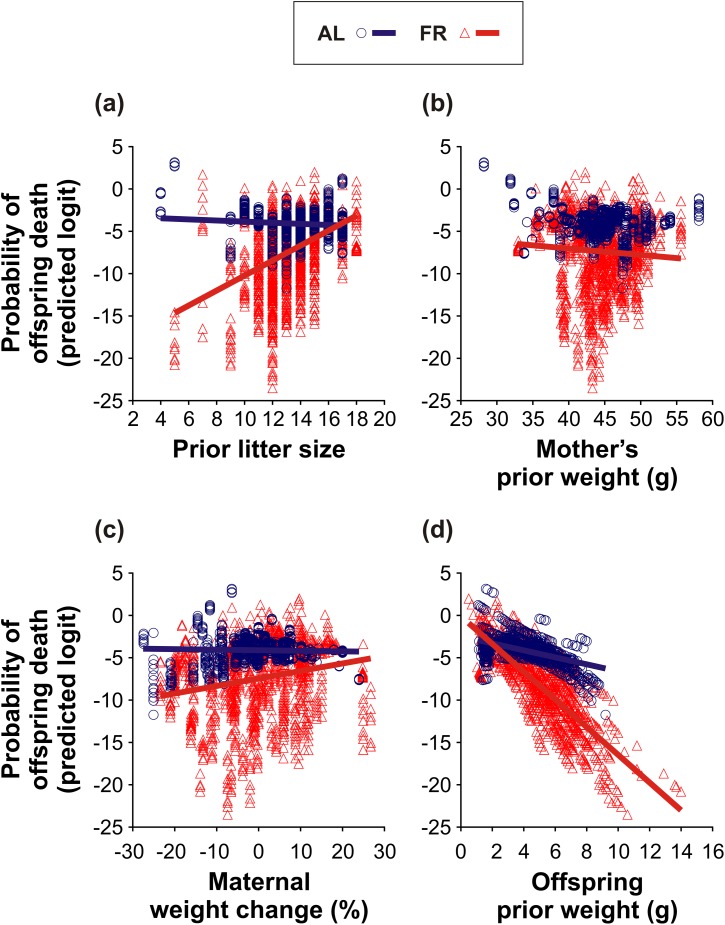
Probability of offspring death. The effect of pre-breeding maternal feeding treatment (*ad libitum*–AL; food restriction–FR) on the relationships between the probability of offspring death and (a) prior litter size, (b) mother’s prior weight, (c) maternal weight change, and (d) offspring prior weight. Only the regression lines of the significant relationships are shown.

**Fig 8:**
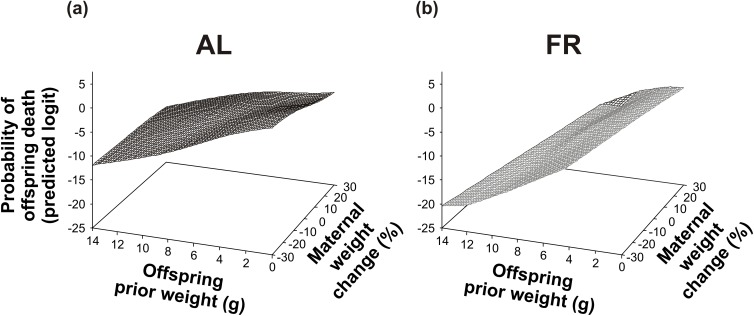
Interaction of maternal weight change with offspring weight. The probability of offspring death depending on the interaction between the maternal weight change and offspring prior weight (a) in the *ad libitum* (AL)-fed mothers, and (b) in the food-restricted (FR) mothers.

The factors that affected the probability of offspring death are listed in Table 5. On average, the probability of offspring death was higher in the AL-fed than in the FR group (logits ± S.E.; AL: -4.21 ± 0.42; FR: -7.08 ± 0.70; Table 5). In addition, offspring survival in the AL-fed and in the FR mice was affected differently by prior litter size (Table 5; Fig 7A), by the mother’s prior weight (Table 5; Fig 7B), by maternal weight change (Table 5; Fig 7C), and by the offspring prior weight (Table 5; Fig 7D). The risk of offspring mortality increased with decreasing size of the litter in the AL-fed group (*t*_626_ = -3.59, *P* < 0.001; Fig 7A), whilst it increased with increasing size of the litter in the FR group (*t*_142_ = -3.71, *P* < 0.001; Fig 7A). In both groups, the risk of offspring mortality tended to increase with the decreasing weight of the mother, however this trend was only significant in the FR mice (AL: *t*_46_ = 0.53, *P* = 0.60; FR: *t*_156_ = 8.31, *P* < 0.0001; Fig 7B). Further, the more weight the mother had lost, the more the risk of offspring mortality increased in the AL-fed group (*t*_198_ = -3.96, *P* < 0.001; Fig 7C), whilst this risk decreased in the FR group (*t*_271_ = 3.58, *P* < 0.001; Fig 7C). In both groups, the probability of offspring death increased with the decreasing weight of the offspring. Nevertheless, this effect was less pronounced in the AL-fed than in the FR group (AL: *t*_868_ = -3.99, *P* < 0.0001; FR: *t*_414_ = -8.61, *P* < 0.0001; Fig 7D). Finally, offspring survival was affected by the interaction between the maternal weight change and offspring prior weight (Table 5; Fig 8). In both groups, the probability of offspring death increased with the increasing weight loss of the mother and with the decreasing weight of the offspring. However, this increase was less severe in the AL-fed group (*t*_579_ = 2.94, *P* < 0.01; Fig 8A) than in the FR group (*t*_933_ = -3.14, *P* < 0.01; Fig 8B).

**Table 5:** Final GzLMM Model with Factors that Affected Probability of Offspring Death.

Fixed effect	df (Num, Den)	*F*-value	*P*-value
Feeding treatment	1, 61.74	42.73	< 0.0001
Prior litter size (Feeding treatment)	2, 249.20	13.34	< 0.0001
Mother’s prior weight (Feeding treatment)	2, 73.89	34.56	< 0.0001
Maternal weight change (Feeding treatment)	2, 229.20	14.40	< 0.0001
Offspring prior weight (Feeding treatment)	2, 583.10	45.05	< 0.0001
Maternal weight change * Offspring prior weight (Feeding treatment)	2, 720.50	9.26	< 0.0001

## Discussion

This is the first study to focus on the role of pre-breeding resource limitations for the optimization of female parental investment during the lactation period. We observed that the exposure of female mice to pre-breeding FR improved the optimization of their litter size. The FR mothers could therefore invest more efficiently in their offspring than the AL-fed mothers. As a consequence of this, the offspring of the FR mothers were in better condition and suffered less from pre-weaning mortality than those of the AL-fed mothers. Our results thus indicate, that pre-breeding FR had wide-ranging long-term effects on parental investment and offspring developmental success (*i*.*e*. increased growth and decreased mortality). These findings correspond to previous studies [35–38] suggesting the potentially beneficial effect of FR on female reproductive fitness. The complex optimization of litter size reflects the *r*-selected life-history, which primarily involves the maximization of offspring numbers in an unpredictable environment. This parental investment strategy may therefore explain why we observed no effects of offspring sex and litter sex ratio during the course of lactation.

The exposure of females to pre-breeding FR improved their parental efforts throughout the whole period of lactation, as indicated by generally larger litters at birth and weaning (see Fig 2). It is important to note that at both these stages the litter size was independent of their weight at mating, despite FR causing a decrease in weight at mating. This may be caused by either one of two reasons. First, the FR mothers might have been in a relatively better condition due to compensatory eating following the period of intermittent fasting (as suggested by [33,39]). Second, they may have increased the size of their litters because of environmental cues indicating a risky environment. The second possibility corresponds to both prior theoretical assumptions [16–19] and empirical evidence [20,40]. Since the FR mice tended to produce daughters, who were lighter and thus less expensive than sons (see [33]), another, not mutually exclusive, explanation may be that they optimized their reproductive costs (as predicted by Myers sex allocation hypothesis: [41]). In addition, in both groups, we observed families with and without litter reduction (see Fig 2). This variability suggests that breeding females “played” at least four different “bet-hedging” strategies (*sensu* “maximization of lifetime reproductive fitness”, p. 2963: [42]). In general, they had to consider whether to “play” either a “safe bet-hedging” or a “risky bet-hedging” strategy, recognized by a small or a large litter at birth, respectively. On the basis that pre-breeding FR of the mothers improved their offspring’s survival during lactation (see Fig 3), we can infer that the “risky bet-hedging” strategy, which encompassed the production of large litters, was more successful in those mice that had encountered resource limitations (as predicted by [19]).

Pre-breeding FR increased the occurrence of litter reduction in the FR mice, but at the same time it also improved the survival of their offspring, as indicated by a lower (see Fig 3) and more selective (size-dependent; Fig 7D) offspring mortality. These results support the conclusions of our previous study [33] and are in accordance with other studies [35,37] that have shown the positive impact of pre-breeding maternal FR on offspring survival. The better condition and survival of the offspring of the FR mice suggest that the FR-improved maternal investment enhanced offspring competitiveness and viability. This effect increased with the age of the offspring (see Fig 4). A breaking point in their development was likely lactation period IV (day 14–17), during which the young mice started to consume solid food [30,43]. In this period, in particular, the smaller offspring could face a decline in milk yield and a rise in calorie expenditure on thermoregulation [44], which may have made them more vulnerable to death due to increased sibling rivalry [25].

Consistent with most previous studies (reviewed in [45]), we observed no active killing of young by their mothers. However, in both groups, we recorded neglected pups lying alone outside of the nest (usually in the corner of the cage), and also partly or completely eaten pups. Thus, although we only observed signs of maternal cannibalism, it is very likely that some of the offspring died due to selective (size-dependent) maternal infanticide. In any event, our results generally indicate that the selective reduction of the litter helped the FR mothers to maintain their own optimum condition, thereby improving the condition and, indirectly, the survival of their remaining offspring (see Figs 5–8). Hence, our findings correspond to the results of Drummond *et al*. [46] who showed that in rabbits, *Oryctolagus cuniculus*, litter reduction improved the growth of the remaining young. Since in the AL-fed mothers litter reduction was, on the contrary, associated with a gradual decline of maternal condition (see Fig 5C) and offspring condition (see Fig 6C), it would seem that the role of litter reduction varied according to the pre-breeding maternal condition. Whereas the FR females continuously adjusted the size of their litters to stabilize the energy burdens of lactation, the litter reduction in the AL-fed females was an inescapable consequence of “maternal optimism” (*i*.*e*. overemphasizing good conditions at the time of breeding, thereby incurring increased reproductive costs during lactation: [26,47]). We can therefore infer that the selective reduction of the litter may have facilitated the FR mice to optimize their inclusive fitness (as predicted by Hamilton’s theory of kin selection: [48]).

A decline in the probability of offspring death with decreasing litter size (see Fig 7A) and with increasing maternal weight (see Fig 7B) in the FR mice is in agreement with theoretical predictions [10,11,24], as well as with previous empirical findings [30,31]. These results show that pre-breeding FR stimulated lactating females to optimize resource allocation according to their actual condition. In contrast, an inverse relationship between the probability of offspring death and litter size in the AL-fed mice (see Fig 7A) supports our assumption of a gradual decline of maternal and offspring condition with increasing numbers of offspring. The FR-induced dichotomy of reproductive tactics may also be partly responsible for the group-specific effect of maternal condition on offspring survival (see Fig 7C). As discussed above, the food intake of lactating mice declines with decreasing litter size [49,50]. Therefore, a lower offspring mortality in lighter FR mice (see Fig 7C) may just reflect a reduction of their litters. On the other hand, a higher offspring mortality in lighter AL-fed mice (see Fig 7C) most likely reflects the selection of large litters [51] and “maternal optimism” [26,47], also as discussed above. All of these effects (see Fig 7A–7C) may have influenced the offspring’s own condition (see Fig 7D). It should be emphasized that unlike the young of the AL-fed mice, the young of the FR mice substantially improved their chances of survival if they had gained weight (during a particular lactation period). Furthermore, their survival improved with an increase in maternal condition (see Fig 8), which suggests that the litter reduction was much more selective (*sensu* “parentally biased favouritism”, p. 381: [52]) in the FR than in the AL-fed mice. Our results thus indicate that the FR mothers had more control over sibling rivalry and their offspring’s development than the AL-fed controls.

As far as we are aware, our study is also the first to document a positive impact of pre-breeding maternal FR on postnatal (pre-weaning) offspring growth. The lower weight gain of the FR mothers also suggests that they expended more energy raising their young than the controls. Our results (see Fig 5A and 5B) indicate that the FR mothers accommodated their food intake to satisfy the actual energetic demands of their offspring [49–51,53]. The AL-fed mothers, on the other hand, did not follow their offspring’s changing demands (see Fig 5B), favouring their own condition over that of their offspring. To the best of our knowledge, a similar transfer of reproductive costs to offspring has only ever been observed in female bighorn sheep, *Ovis canadensis* [54]. The FR-modified relationship between maternal and offspring condition (see Fig 6B) best reflects this dichotomy of female reproductive tactics. We observed that as the maternal weight loss increased, the offspring of the FR mice lost weight significantly less rapidly than those of the AL-fed controls. An alternative, not mutually exclusive, explanation of the maternal effect on offspring weight is that the size-dependent mortality of the marginal offspring improved the growth of the remaining, heavier siblings.

One could argue that the positive association between offspring growth and litter size (see Fig 6A) contradicts the classic offspring size-number trade-off [8,13]. Nevertheless, because the females most likely decreased their food intake along with the reduction of the litter (see Fig 5A), it is possible that this relationship stemmed from the reduction in resource allocation [50,55,56]. An alternative explanation for this relationship may be the selection of large litters, which is accompanied by increased milk production and offspring growth [51,57,58]. Again, we saw that this effect was more intense for the offspring of the FR mothers.

We assume that the FR mothers expended greater parental investment than the AL-fed mothers, since they had coped with pre-breeding FR and passed through strict selection for successful reproduction [33]. Hence, the FR females may actually have been in a better condition than the controls over the whole lactation period [33,39]. This improvement in their condition may be the result of compensatory food intake induced by the combined effect of intermittent feeding and food deprivation before breeding. In addition, an extended period of fasting followed by *ad libitum* eating could have triggered a cascade of biological processes that improved their reproductive performance (including ovulation rate, implantation rate, embryonic survival, litter size, milk production, milk composition, and offspring development). A number of previous studies have reported that the FR-induced weight loss promotes feeding behaviour that results in a rapid regaining of weight to a specific body weight “set-point” (for review, see [59]). The physiological mediator of this behaviour is mainly the orexigenic hormone ghrelin, which stimulates appetite by acting on the hypothalamic arcuate nucleus [60]. This region of the brain is also the target of the anorexigenic hormone leptin, whose production increases with increasing weight gain [61], and which can promote maternal investment [62]. It can therefore be inferred that as the adiposity of the FR females increased, leptin levels raised and intensified their maternal care. The ghrelin-leptin system may thus have maintained their energy balance. The weight regain-elevated leptin levels may also have improved the survival of the FR mice’s young, which French *et al*. [62] observed in the Siberian hamsters, *Phodopus sungorus*. It also cannot be excluded that the improved postnatal somatic growth and survival resulted from specific epigenetic processes induced by the FR-altered maternal physiology and behaviour [63–65].

The ultimate reason for the FR-induced divergence of reproductive optima may be a state-dependent life-history tactic, which involves the adjustment of offspring phenotypes to the selective pressures that operate in unpredictable environments [66]. Hence, anticipating changing food availability, the optimal reproductive tactic may be either a complete inhibition of reproduction or an increase of parental investment, leading to the production of larger litters with strong, competitively successful offspring. This enormous plasticity of the reaction norm may provide the key to the ecological success of the house mouse. It also suggests that environmental uncertainty could play a critical role in its adaptive evolution [67].

## Conclusions

**Fig 9:**
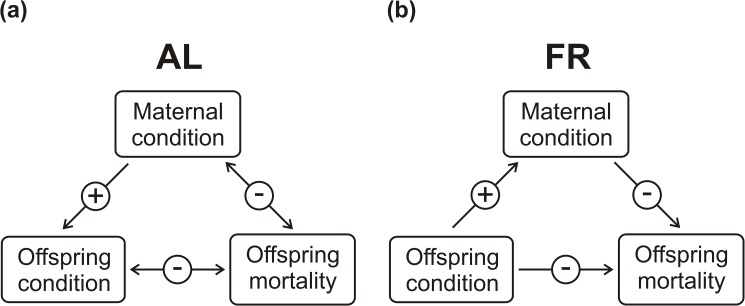
Mechanisms of fitness optimization. The impact of pre-breeding maternal feeding treatment (a: *ad libitum*–AL; b: food restriction–FR) on the relationships between maternal condition (mother’s prior weight, maternal weight change), offspring condition (offspring prior weight, offspring weight change), and offspring mortality (probability of offspring death, litter biomass change). The arrows indicate the directions of the significant effects of particular relationships. Positive and negative relationships are respectively denoted with + and–. The scheme demonstrates that: (1) offspring condition was only positively associated with maternal condition in the AL-fed mice; (2) maternal condition was only positively associated with offspring condition in the FR mice; and (3) offspring mortality decreased with increasing maternal and offspring condition in both groups, but only in the AL-fed mice did their condition decline with increasing offspring mortality.

Our study shows that pre-breeding FR stimulated lactating mice towards effective parental investment. This resulted in a dichotomy of reproductive optima (see Fig 9 – summarizing diagram). Exposing female mice to unpredictable food availability induced highly selective litter reduction that improved their fitness prospects. In contrast, the control females fed *ad libitum* produced litters of super-optimal size and thus incurred higher reproductive costs, including a greater reduction of their litters. We believe that our findings may have important implications for the understanding of the mechanisms of life-history evolution, as well as potentially for animal breeding and for research into the negative effects of obesity on reproduction. To fully evaluate the significance of these findings, future research should: (1) examine the influence of environmental uncertainty on parental fitness in natural populations of mammals; (2) determine whether pre-breeding resource limitations can benefit caring parents and their dependent offspring in a changeable environment; and (3) identify the genes and pathways involved in the optimization of parental investment.

## Supporting information

S1 Appendix SAS codes for the analyses. (PDF)

S1 Dataset Dataset used in the analyses. S1 Dataset file contains five worksheets with the following data subsets: (1) maternal and litter traits, (2) dynamics of offspring mortality, (3) maternal weight change, (4) offspring weight change, and (5) probability of offspring death. The maternal and litter traits were used to determine the impact of pre-breeding FR on birth litter size, weaning litter size, and total litter reduction. The dynamics of offspring mortality were used to assess the effect of pre-breeding FR on offspring mortality in particular lactation periods. The maternal weight change, offspring weight change, and probability of offspring death were used to evaluate the influence of pre-breeding FR on the relationships between maternal and offspring condition, as well as offspring mortality, during lactation periods. Each of these last three subsets also contains the predicted values of the dependent variable.
(XLSX)
