# Correction: Pre-breeding food restriction promotes the optimization of parental investment in house mice, *Mus musculus*

**DOI:** 10.1371/journal.pone.0178804

**Published:** 2017-05-26

**Authors:** Adam Dušek, Luděk Bartoš, František Sedláček

There are errors in the reference list. Reference 58 should have been listed as Reference 39. The correct References 39 to 58 are:

**39**. Meikle DB, Thornton MW. Premating and gestational effects of maternal nutrition on secondary sex ratio in house mice. J Reprod Fertil. 1995; 105: 193–196.

**40**. Mappes T, Ylönen H. Reproductive effort of female bank voles in a risky environment. Evol Ecol. 1997; 11: 591–598.

**41**. Myers JH. Sex ratio adjustment under food stress: maximization of quality or numbers of offspring? Am Nat. 1978; 112: 381–388.

**42**. Olofsson H, Ripa J, Jonzen N. Bet-hedging as an evolutionary game: the trade-off between egg size and number. Proc Biol Sci. 2009; 276: 2963–2969.

**43**. König B, Markl H. Maternal care in house mice. I. The weaning strategy as a means for parental manipulation of offspring quality. Behav Ecol Sociobiol. 1987; 20: 1–9.

**44**. König B, Riester J, Markl H. Maternal care in house mice (*Mus musculus*). II. The energy cost of lactation as a function of litter size. J Zool. 1988; 216: 195–210.

**45**. Weber EM, Olsson IAS. Maternal behaviour in *Mus musculus sp*.: an ethological review. Appl Anim Behav Sci. 2008; 114: 1–22.

**46**. Drummond H, Vázquez E, Sánchez-Colón S, Martínez-Gómez M, Hudson R. Competition for milk in the domestic rabbit: survivors benefit from littermate deaths. Ethology. 2000; 106: 511–526.

**47**. McNamara JM, Trimmer PC, Eriksson A, Marshall JAR, Houston AI. Environmental variability can select for optimism or pessimism. Ecol Lett. 2011; 14: 58–62.

**48**. Hamilton WD. The genetical evolution of social behaviour. I. II. J Theor Biol. 1964; 7: 1–52.

**49**. Aloise King ED, Garratt M, Brooks R. Manipulating reproductive effort leads to changes in female reproductive scheduling but not oxidative stress. Ecol Evol. 2013; 3: 4161–4171.

**50**. Johnson MS, Thomson SC, Speakman JR. Limits to sustained energy intake I. Lactation in the laboratory mouse *Mus musculus*. J Exp Biol. 2001; 204: 1925–1935.

**51**. Rauw WM, Luiting P, Beilharz RG, Verstegen MWA, Vangen O. Selection for litter size and its consequences for the allocation of feed resources: a concept and its implications illustrated by mice selection experiments. Livest Prod Sci. 1999; 60: 329–342.

**52**. Lessells CM. Parentally biased favouritism: why should parents specialize in caring for different offspring? Philos Trans R Soc Lond B Biol Sci. 2002; 357: 381–403.

**53**. Hammond KA, Diamond J. An experimental test for a ceiling on sustained metabolic rate in lactating mice. Physiol Zool. 1992; 65: 952–977.

**54**. Martin JGA, Festa-Bianchet M. Bighorn ewes transfer the costs of reproduction to their lambs. Am Nat. 2010; 176: 414–423.

**55**. Schroderus E, Koivula M, Koskela E, Mappes T, Oksanen T, *et al*. Can number and size of offspring increase simultaneously?—a central life-history trade-off reconsidered. BMC Evol Biol. 2012; 12: 1–7.

**56**. van Noordwijk AJ, de Jong G. Acquisition and allocation of resources: their influence on variation in life history tactics. Am Nat. 1986; 128: 137–142.

**57**. Schubert KA, de Vries G, Vaanholt LM, Meijer HAJ, Daan S, et al. Maternal energy allocation to offspring increases with environmental quality in house mice. Am Nat. 2009; 173: 831–840.

**58**. Skjervold H. The effect of foetal litter size on milk yield: cross fostering experiments with mice. J Anim Breed Genet. 1977; 94: 66–75.
