# Supplementary material for: Pre-breeding food restriction promotes the optimization of parental investment in house mice, Mus musculus
Source: PLoS One. 2017 Mar 22;12(3):e0173985. doi: 10.1371/journal.pone.0173985 (PMC5362082; doi:10.1371/journal.pone.0173985)
Supplement: S1 Appendix — (PDF) [file pone.0173985.s001.pdf]

S1 Appendix. SAS codes for the analyses.

T-test for effect of feeding treatment on maternal weight at mating

```
proc ttest
data=&File
alpha=0.05
H0=0
CI=EQUAL;
class feeding_treatment;
var maternal_weight_at_mating;
title "PROC TTEST for maternal weight at mating";
run;
```

T-test for effect of feeding treatment on maternal delivery weight

```
proc ttest
data=&File
alpha=0.05
H0=0
CI=EQUAL;
class feeding_treatment;
var maternal_delivery_weight;
title "PROC TTEST for maternal delivery weight";
run;
```

Final GLMM model with factors that affected birth litter size

```
%let Var=birth_litter_size;
%let Class=experimental_trial feeding_treatment litter_reduction;
%let Random=experimental_trial;
proc mixed data=&File method=reml covtest;
class &Class;
model &Var=feeding_treatment litter_reduction(feeding_treatment)
maternal_weight_at_mating(feeding_treatment) / ddfm=kr outp=pred solution
e3 htype=1,2,3;
random &Random;
title "PROC MIXED for birth litter size";
lsmeans feeding_treatment / pdiff ADJUST=TUKEY;
lsmeans litter_reduction(feeding_treatment) / pdiff ADJUST=TUKEY;
ods output lsmeans=lsmeans;
run;
```

Final GLMM model with factors that affected weaning litter size

```
%let Var=weaning_litter_size;
%let Class=experimental_trial feeding_treatment litter_reduction;
%let Random=experimental_trial;
proc mixed data=&File method=reml covtest;
class &Class;
model &Var=feeding_treatment litter_reduction(feeding_treatment)
maternal_weight_at_mating(feeding_treatment)
maternal_weight_change_at_weaning(feeding_treatment) / ddfm=kr outp=pred
solution e3 htype=1,2,3;
random &Random;
title "PROC MIXED for weaning litter size";
lsmeans feeding_treatment / pdiff ADJUST=TUKEY;
lsmeans litter_reduction(feeding_treatment) / pdiff ADJUST=TUKEY;
ods output lsmeans=lsmeans;
run;
```

χ^2 contingency test for effect of feeding treatment on total litter reduction

```
proc freq data=&File;
tables litter_reduction*feeding_treatment / exact chisq cellchi2 expected
nopercent CMH1 CMH2;
title "PROC FREQ for total litter reduction";
run;
```

χ^2 contingency test for effect of feeding treatment on dynamics of offspring mortality

```
proc freq data=&File;
tables lactation_period*litter_reduction*feeding_treatment / exact chisq
cellchi2 expected nopercent CMH1 CMH2;
title "PROC FREQ for dynamics of offspring mortality";
run;
```

Final GLMM model with factors that affected maternal weight change

```
%let Var=maternal_weight_change;
%let Class=experimental_trial feeding_treatment lactation_period
ID_of_mother ID_of_offspring;
%let Random=experimental_trial;
%let Repeated=lactation_period;
proc mixed data=&File method=reml covtest;
class &Class;
model &Var=feeding_treatment prior_litter_size(feeding_treatment)
offspring_weight_change(feeding_treatment)
litter_biomass_change(feeding_treatment) / ddfm=kr outp=pred solution e3
htype=1,2,3;
random &Random;
repeated &Repeated / type=cs subject=ID_of_offspring(ID_of_mother) r rcorr;
title "PROC MIXED for maternal weight change";
lsmeans feeding_treatment / pdiff ADJUST=TUKEY;
ods output lsmeans=lsmeans;
ods output covparms=cov rcorr=corr;
run;
```

Final GLMM model with factors that affected offspring weight change

```
%let Var=offspring_weight_change;
%let Class=experimental_trial feeding_treatment lactation_period
ID_of_mother ID_of_offspring;
%let Random=experimental_trial ID_of_mother;
%let Repeated=lactation_period;
proc mixed data=&File method=reml covtest;
class &Class;
model &Var=feeding_treatment prior_litter_size(feeding_treatment)
maternal_weight_change(feeding_treatment)
litter_biomass_change(feeding_treatment) / ddfm=kr outp=pred solution e3
htype=1,2,3;
random &Random;
repeated &Repeated / type=cs subject=ID_of_offspring(ID_of_mother) r rcorr;
title "PROC MIXED for offspring weight change";
lsmeans feeding_treatment / pdiff ADJUST=TUKEY;
ods output lsmeans=lsmeans;
ods output covparms=cov rcorr=corr;
run;
```

Final GzLMM model with factors that affected probability of offspring death

```
%let Var=offspring_mortality;
%let Class=experimental_trial feeding_treatment lactation_period
ID_of_mother ID_of_offspring;
%let Random=experimental_trial ID_of_mother;
%let Subject=ID_of_offspring(ID_of_mother);
proc glimmix data=&File plots=boxplot plots=pearsonpanel;
class &Class;
model &Var=feeding_treatment prior_litter_size(feeding_treatment)
mother_prior_weight(feeding_treatment)
maternal_weight_change(feeding_treatment)
offspring_prior_weight(feeding_treatment)
maternal_weight_change*offspring_prior_weight(feeding_treatment) / dfm=kr
dist=binary link=logit oddsratio solution;
random &Random;
random _residual_ / subject=&Subject type=ar(1);
title "PROC GLIMMIX for probability of offspring death";
lsmeans feeding_treatment / diff ADJUST=TUKEY oddsratio plots=diffplot
plots=meanplot;
output out=pred pred=pred lcl=lower;
run;
```
